# Book Reviews

**Published:** 1897-12

**Authors:** 


### *BOOK REVIEWS.*

A System of Medicine. Edited by T. Clifford Allbutt, M.A., M.D., P.R.C.P., etc. Vol. IV. Illustrated. pp. 1001. MacMillan & Co., 66 Fifth Ave., New York.

We had recently the pleasure of reviewing the previous volume of this system, and the words of praise used in reference to it might be repeated in connection with this. It considers General Diseases of Obscure Origin and Diseases of Alimentation and Excretion, including those of the Stomach, Subphrenic Abscess, Diaphragmatic Hernia, Abdominal Diagnosis from Gynecological standpoint, Enteroptosis, Diseases of the Peritoneum and of the Bowels. Dr. Allbutt has himself dealt with Mountain Sickness and Dilatation of the Stomach; Dr. Cheadle with Rickets, and in conjunction with Dr. Church, with Acute Rheumatism; Dr. Archibald Garrod with Chronic, Muscular and Gonorrheal Rheumatism, and along with Dr. Kent Spender, with Rheumatoid Arthritis, which, as it is found in children, is described by Dr. Sill. Dr. Bowlby takes up Osteomalacia, Osteitis Deformans, Acromegaly and Hypertrophic Pulmonary Osteo-Arthropathy; Sir William Roberts, Gout; Dr. Saundby, Diabetes Mellitus; the late Dr. Ralfe, Diabetes Insipidus, and with Dr. Soltan Fenwick, who also treats of Dyspepsia in Childhood, the General Pathology of Digestion; Dr. Howship Dickinson, Lardaceous Disease; Dr. Ross Bradford, the General Pathology of Secretion; Dr. Cobbett, Shock and Collapse; Dr. W. A. Wills, Diseases of the Mouth; and Dr. Rolleston, Diseases of the Esophagus. To Dr. Lauder Brunton have fallen Dyspepsia, the Physiology of Fecal Evacuation, Diarrhea, and along with Dr. Leith, Gastritis; to Dr. Stocker, Sea-sickness; to Dr. Dreschfeld, Ulcer of the Stomach and Duodenum; to Dr. Hale White, Tumors of the Stomach and Diseases of the Colon; to Dr. Lee Dickenson, Subphrenic Abscess and Diaphragmatic Hernia; to Dr. Playfair, Abdominal Diagnosis from a Gynecological Standpoint; to Mr. Treves, Enteroptosis, Acute Peritonitis, Intestinal Obstruction and Perityphlitis; to Dr. Allchin, Acute Peritonitis of Undetermined Nature, Chronic Peritonitis, Tubercle of the Peritoneum and New Growths of the Peritoneum; to Dr. Eustace Smith, Diarrheas of Children; to Dr. Manson, Sprue or Psilosis, and to Dr. Allingham, the Differential Diagnosis of Diseases of the Anus and Rectum. This is a list which speaks for itself. The names of all or most of these authors are well-known in this country, and betoken a work of the highest excellence and of the greatest usefulness. The volume deals with many common and important every-day diseases, as well as with some others which are rarely seen, and are therefore of less general interest, but on the whole it may truthfully be asserted that it is a book which every one practicing general medicine, and markedly those having a special interest in abdominal surgery, should carefully study. Dr. Treves's account of perityphlitis is well worthy of perusal at the present time on account of the great interest shown here in this eminently American disease. It would be invidious to pick out as better than the others any of these contributions, all of which appear to us to be of a very high order of merit. The publishers have as usual done their work well. A. W. S.

---

AN EPITOME OF THE HISTORY OF MEDICINE. By Roswell Park, A.M., M.D., Professor of Surgery in the Medical Department of the University of Buffalo, etc. Illustrated with Portraits and other Engravings. One Volume, Royal Octavo, pages xiv–348. Extra cloth. Beveled edges, $2 net. The F. A. Davis Co., Publishers, 1914 and 1916 Cherry street, Philadelphia.

This interesting work is based upon a series of lectures which were delivered two or three years ago in the Medical Department of the University of Buffalo. The "subject-matter of these lectures has been rearranged, enlarged and edited in order to make it more presentable for easy reading and reference."

It is quite true, as Dr. Park says in his preface, that "the history of medicine has been sadly neglected in our medical schools." The evolution of medical and surgical principles and of the successive steps which have led up to great discoveries and achievements in our art are certainly important enough to be duly recognized in our curricula. And sooner or later the history of medicine will be taught in all our schools.

In presenting his subject the author has adopted the arrangement of Renouard; dividing the past into three ages, the *Age of Foundation*, beginning in myth, and ending with the death of Galen, A. D. 200; the *Age of Transition*, ending with the revival of letters, A. D. 1400; and the *Age of Renovation*, from the latter date to the present day. Separate chapters have been given to the "History of Medicine in America," the "History of Anesthesia," the "History of Antiseptics," and an "Epitome of the History of Dentistry."

This book aught to be in every medical library. It tells of the thoughts and labors of the past masters in the science and art of studying and treating disease; of that long line of immortals, the Asclepiadæ, Hippocrates, Galen, Paulus Egineta, Avicenna, Maimonides, Roger of Salerno, Guy de Chauliac, Linacre, Vesalius, Fallopius, Ambroise Paré, Harvey, Malpighi, Sydenham, Boerhaave, Cullen, van Swieten, Bichat, Desault, Richter, the Hunters, the Bells, Jenner, Bernard, Broussais, Louis, Henle, Virchow, Koch, Lister, Pasteur, Laennec, Dupuytren, Nelaton, Velpeau, Astley Cooper, Simpson, Billroth, McDowell, Sims, and so on through the illustrious list.

To write an epitome of medical history is necessarily a difficult task; but Dr. Park has accomplished it with credit to himself and to the great delight of those who will read his book.

---

CUTANEOUS MEDICINE—A SYSTEMATIC TREATISE ON THE DISEASES OF THE SKIN. By Louis A. Duhring, M.D., Professor of Diseases of the Skin in the University of Pennsylvania, etc., etc. Part II. Classification, Anemias, Hyperemias, Inflammations. Illustrated. Philadelphia: J. B. Lippincott Co.

This is the second part of what promises to be the American Classic upon Diseases of the Skin. Part I. dealt with the principles of Dermatology, and is the most valuable work of the kind with which we are acquainted. Part II. takes up the practice of Dermatology. A very great defect in the two parts which we have seen is the utter absence of a table of contents and index, which certainly would have been proper in each volume.

Classification is based upon the clinical appearances, combined with the pathology. Classification of skin diseases is a difficult and complex matter, and, aside from the well-known diseases, is much the result of individual judgment, and the one adopted in this work will meet its share of opposition. But, if we must have a classification, Duhring's will not be the worst. The text is full, and drawn both from the author's large experience and the writings of the world's best authorities.

Foot-notes are numerous and present many practical points not brought out in the body of the articles. The illustrations are numerous and for the most part good. Histological drawings are excellent.

He retains many of the disease names present in his old work—such as Impetigo and Ecthyma. It seems to us that this is not quite up to modern views—many of these pustular diseases belonging properly under the common head of infectious, pus diseases, and that the pus organisms should be given full credit, as is now given the tubercle bacillus in many formerly distinct diseases. The work, when complete, is sure to become a standard, but we can only wish that the author had anticipated the future by condensing the number and variety of skin diseases into a smaller list, wholly according to a common pathology and treatment.

M. B. H.

---

TEXT-BOOK OF PRACTICAL THERAPEUTICS, with especial reference to the application of Remedial Measures to Disease and their Employment upon a Rational Basis. By Hobart Amory Hare, M.D., Professor of Therapeutics and Materia Medica in the Jefferson Medical College, Philadelphia, etc. With special chapters by Drs. George E. de Schweinitz, Edward Martin and Barton C. Hirst. Sixth edition, thoroughly revised and largely rewritten. In one octavo volume of 756 pages. Cloth, $3.75; leather, $4.75, Lea Brothers & Co., Publishers, Philadelphia and New York, 1897.

The measure of success attained by the author in his effort to provide the physician or undergraduate student of medicine with a reliable guide in the study of therapeutics and the application of remedial agents in the cure of disease is indicated by the call for another revision of this volume, the sixth within a period of seven years. No small part of the approval earned may be justly due to the original plan upon which the volume is constructed, being divided into two sections, in the first of which the author deals with remedial agents, both medicinal and non-medicinal, while in the second he presents a description of the various diseases, their symptoms, complications, treatment, etc.

The previous edition has been practically rewritten, and such new measures as have proved useful and reliable have been given due consideration. Four pages are devoted to the antitoxin treatment of diphtheria, which, the author thinks, "supplants all others in efficacy and usefulness." Under *gonorrhea*, however, no mention is made of irrigation with permanganate of potash solutions, which has given such happy results in the hands of many surgeons. Among the new drugs receiving mention are: Eucaine, formaldehyde, kola, methyl blue, naphthol, nuclein, pyrogallol, scopolamine, suprarenal gland, thymus and thyroid glands, trichloracetic acid, trional, etc.; and among the new therapeutic measures, not drugs, are: Enteroclysis, hypodermoclysis, lavage, etc.; and a useful chapter has been added on mineral springs and climates.

This is the best book on practical materia medica and therapeutics for the student that we have ever read. It should continue to enjoy its well-earned popularity.

---

TEXT-BOOK OF THE PRACTICE OF MEDICINE. By James M. Anders, M.D., LL.D., Professor of the Practice of Medicine and Clinical Medicine in the Medico-Chirurgical College, Philadelphia; Attending Physician to the Medico-Chirurgical and Samaritan Hospitals, etc. Illustrated. pp. 1285. W. B. Saunders, 925 Walnut St., Philadelphia. Price, $5.50 net.

The author of this volume has aimed to produce a work which would reflect the present state of our knowledge of the practice of medicine in general and of the diagnosis and treatment of disease in particular. The historical development of the subjects treated has been either briefly mentioned or intentionally omitted. Exploded views and abandoned therapeutic measures are not given. In this respect this book will be found to differ from revised editions of some other works which have been kept before the profession for many years. Evidences are numerous that the author has adhered faithfully to his purpose and produced a strictly practical, scientific and up-to-date work.

Due prominence has been given to bacteriology under the special etiology of diseases. In fifty-six important instances the essential facts of differential diagnosis have been arranged in diagnostic tables; this will be of considerable help, particularly to students. A large number of formulæ and prescriptions have been introduced in the text, which the author in a large experience has tested and proven to be possessed of real therapeutic importance. Prescriptions are written in both the English and metric systems.

One cannot read this work without receiving a most favorable impression. It is well written, and nowhere, as far as we know, have the important facts of the symptoms, pathology, diagnosis and treatment of disease been narrated in a more pleasing and instructive manner. The book merits a generous reception from both students of medicine and veteran practitioners.

---

INCOMPATIBILITIES IN PRESCRIPTIONS. For Pharmacists and Physicians. By E. A. Ruddiman, Ph.M., M.D., Adjunct Professor of Pharmacy and Materia Medica in Vanderbilt University, Nashville. 267 pages. Price, $2.00. John Wiley & Sons, 53 East Tenth St., New York.

"The busy prescriptionist is frequently at a loss to know what takes place in the prescription he is filling, and does not have the time nor books necessary to look up the change which he has noticed. The object of the first part of this book is to present to him in a convenient and condensed form the more common incompatibilities. The substances treated of are arranged in alphabetical order of their Latin names, except in case of some of the newer remedies. The second object of the writer is to furnish the student of pharmacy with a list of incompatible prescriptions in such form that he may find out for himself what the trouble is, and the best means of avoiding or overcoming it."

Physicians often know too little about the combinations which they prescribe. Druggists generally know very little more; sometimes less. This book contains numerous suggestions as to the proper construction and compounding of prescriptions that cannot fail to be of value to both prescriber and dispenser. It is the most complete work on incompatibilities which we have seen. Physicians who to some extent dispense their own medicines and physician-druggists will find this book particularly helpful.

---

THE MEDICAL NEWS VISITING LIST FOR 1898. Weekly (dated, for 30 patients); Monthly (undated, for 120 patients per month); Perpetual (undated, for 30 patients weekly per year); and Perpetual (undated, for 60 patients weekly per year), $1.25. Thumb-letter Index, 25 cents extra. Philadelphia and New York: Lea Brothers & Co.

A Visiting List is an indispensable convenience for the active practitioner. Its carefully adapted blanks enable him at once to note clinical details of every-day work, as well as charges and receipts, and to unburden his memory from what can better be carried on paper. It also furnishes him with a legal record necessary for the collection of delinquent bills. Prominent among the many books of this nature stands the *Medical News Visiting List.* Its blank pages are arranged to classify and record memoranda and engagements of every description occurring in the practice of the physician, surgeon or obstetrician. The *Medical News Visiting List* is issued in four styles, adapted to any system of records and any method of keeping professional accounts. It is durably and handsomely bound in the size of a wallet for the pocket. When desired a Ready Reference Thumb-letter Index is furnished which is peculiar to this list and an economizer of time.

---

THE TONIC TREATMENT OF SYPHILIS. By E. L. Keyes, A.M., M.D., Late Professor of Dermatology, Syphilology and Genito-Urinary Surgery, Bellevue Hospital Medical College; Consulting Surgeon to Bellevue Hospital. New York: D. Appleton & Co.

This is a revised edition of the work issued by Dr. Keyes in 1877. The present edition is the result of the doctor's own work since 1876, at which time the theory of the tonic mercurial treatment of syphilis was advanced by him.

He says his meaning has been misunderstood. That by "tonic" he has not meant the use of mercury as a tonic simply, but for its special tonic effect in syphilis. His idea is to use mercury in such doses, constantly, in syphilitic cases that the disease is favorably influenced by the drug, and yet the drug does no injury.

At times, he admits, it is proper to increase the dose of mercury. He goes very thoroughly into the discussion of the treatment of syphilis, and describes the methods of ascertaining the favorable effect of tonic doses of mercury in increasing the number of the red-blood corpuscles. As is characteristic of Keyes' writings, the book is most readable. M. B. H.

---

THE LIVING AGE FOR 1898.

In another column will be found a prospectus of this standard periodical. Founded by Eliakim Littell in 1844, it has steadily maintained the reputation gained with its earliest issues of being the most complete representative of foreign thought as expressed by its greatest exponents. It is to-day a faithful reflection of almost all that is substantial and truly valuable in the passing literature of the world, embracing as it now does in its Monthly Supplement, American as well as foreign literature.

While its pages show the same wise and judicious discrimination which has ever characterized its editorial management, the scope of the magazine has been widened, its size increased and its price reduced, so that increasing years seem only to add to its vigor and value.

To those whose means are limited it must meet with especial favor, for it offers them what could not otherwise be obtained except by a large outlay. Intelligent readers who want to save time and money will find it invaluable.

*The Living Age* is published weekly, and the price is now but $6.00 a year. To all new subscribers for 1898 are offered free the eight numbers of 1897, containing the opening chapters of the new serial, " With All Her Heart," described in the prospectus.

